# Assessment of New Strategies to Improve the Performance of Antimicrobial Peptides

**DOI:** 10.3390/nano12203691

**Published:** 2022-10-20

**Authors:** Lin Wang, Hang Liu, Xinsong Li, Chen Yao

**Affiliations:** School of Chemistry and Chemical Engineering, Southeast University, Nanjing 211189, China

**Keywords:** antimicrobial peptides, lipoic acid, biofilm, crosslink, nanoparticles, phenethylamine

## Abstract

In this research, we constructed a novel engineered tripeptide modified with lipoic acid (LA-RWR), followed by crosslinking of lipoic acid to form nanoparticles (c-LA-RWR). LA-RWR was also modified with phenethylamine (PEA) on the C-terminus to achieve better antibacterial activities. The as-prepared c-LA-RWR and LA-RWR-PEA were effective against *E.* *coli*, *S.* *aureus*, *C.* *albicans*, and methicillin-resistant *Staphylococcus aureus*, with minimum inhibitory concentration values ranging from 2 to 16 µg/mL, which greatly improved the performance of LA-RWR. Similar antibacterial activities were demonstrated in anti-biofilm activity; there was no matter on the biofilm that was already established or forming. Moreover, c-LA-RWR/LA-RWR-PEA remarkably induced cytoplasmic membrane depolarization and outer membrane permeabilization, resulting in varying degrees of damage to the bacterial morphology, which were consistent with the results obtained via electron microscopy. Thus, our results show that c-LA-RWR/LA-RWR-PEA exhibited excellent efficacy against a variety of microorganisms with good biosafety, providing new strategies by which to improve the performance of antimicrobial peptides.

## 1. Introduction

Antimicrobial peptides (AMPs) are an inherent part of an organism’s intrinsic immune system [1,2,3] with a unique membrane-active antibacterial mechanism that does not easily lead to antibiotic resistance tendencies [4,5]. AMPs provide the main defense against bacteria, fungi, viruses, and some multi-drug-resistant bacteria [6,7]. Due to their electropositive and hydrophobic properties, AMPs are endowed with the ability to bind and disrupt cell membranes. Whereas bacterial cell membranes are made up of negative charges, mammalian cell membranes are mainly composed of lipids with zero net charges [8]. In view of the different characteristics of membrane composition, cationic AMPs selectively target negatively charged bacterial membranes through electrostatic interactions, where hydrophobic domains are inserted into the cell membrane to disrupt the integrity of the membrane and ultimately result in death [1,9,10,11].

Despite the great advantages of AMPs, low protease stability and the short duration of activity in clinical applications have hindered their further development [2,12], which has led researchers to focus on synthetic ultrashort peptides [9]. The design of synthetic short peptides is often based on the structure–activity relationship of natural AMPs [11]. However, most direct active fragments are not active enough to be used as therapeutic agents, and further chemical structural modification is necessary [13]. A number of strategies have been used to improve the antimicrobial activities of engineered ultrashort peptides, including amino acid replacement [14,15], modification of the peptide end [16,17], cyclization, and conjugation to polymers or macromolecules [14]. Among these, the modification of hydrophobic alkyl chains at the N/C-terminus of peptides is one of the most common methods for designing engineered antimicrobial lipopeptides, which can also enhance the stability in proteases. Chen et al. developed a new antimicrobial peptide, Chol-37(F34-R), based on PMAP-37(F34-R), via N-terminus cholesterol modification; this had significant advantages such as high antimicrobial activity, high anti-biofilm activity, and low toxicity [18]. Elzbieta and co-workers proved that the binding of the KR12-NH_2_ peptide to C_4_-C_14_ fatty acid chains showed enhanced antimicrobial activities with high potency against plankton and the eradication of bacterial biofilms at low concentrations [19].

Lipoic acid (LA) is an endogenous compound containing disulfide bonds, and is often used as a hydrophobic ligand-modifying drug that can reach any cell in the body and improve overall antibacterial efficacy [20,21,22]. The disulfide bond can be decomposed via interaction with the reducing substance to form a sulfhydryl group, and then the liberated sulfhydryl groups can be crosslinked with each other to form a new disulfide bond. Phenethylamine (PEA) is an aromatic amine that can greatly degrade microorganisms when present in high concentrations because of its hydrophobic properties [23,24]. Xie et al. studied the inhibitory effect of α-phenethylamine on activated sludge and luminescent bacteria and found that its inhibitory effect was greater than that of fosfomycin, an efficient broad-spectrum antibiotic [23].

AMPs containing arginine (R) and tryptophan (W) have been reported with good antibacterial activity [3,25]. In this research, lipopeptide LA-RWR was first developed by modification of LA on the N-terminus of tripeptide RWR. To improve the antibacterial performance of LA-RWR, it was modified using two strategies: one was to crosslink lipoic acid to form nanoparticles (c-LA-RWR), and the other was to modify the C-terminus with PEA to obtain LA-RWR-PEA. The inhibitory effects of c-LA-RWR/LA-RWR-PEA on planktonic bacteria and bacterial biofilms were tested, and antibacterial mechanisms, including membrane depolarization and outer membrane permeability, were further investigated. In addition, the hemolytic activity and the in vitro cytotoxicity of c-LA-RWR/LA-RWR-PEA were evaluated to verify its safety for further applications.

## 2. Materials and Methods

### 2.1. Materials

The test strains *Escherichia coli* ATCC 25922 (*E.* *coli*), *Escherichia coli* DC2 ATCC 8739 (*E. coli* DC2), *Staphylococcus aureus* ATCC 29213 (*S.* *aureus*), *Candida albicans* ATCC10231 (*C.* *albicans*), and methicillin-resistant *Staphylococcus aureus* ATCC43300 (MRSA) were obtained from Shanghai Bioresource Collection Center, SHBCC (Shanghai, China). Luria-Bertani (LB) broth, trypticase soy broth (TSB), Sabouraud dextrose broth, and Mueller–Hinton broth (MHB) were purchased from Hopebiol (Qingdao, China). Alpha-lipoic acid (LA), phenethylamine (PEA), methanol, glacial ether, 3,3-dipropylthiodicarbocyanine iodide (diSC_3_5), N-phenyl-1-naphthylamine (NPN), crystal violet, dimethyl sulfoxide (DMSO), methyl-thiazolyl diphenyl-tetrazolium bromide (MTT), and other chemicals were purchased from Aladdin Co. (Shanghai, China). Mouse fibroblast cells (L929 cells), hamster kidney cells (BHK-21 cells), and newborn calf serum were obtained from KeyGEN BioTECH (Nanjing, China).

### 2.2. Peptide Synthesis and Purification

**Figure A1:**
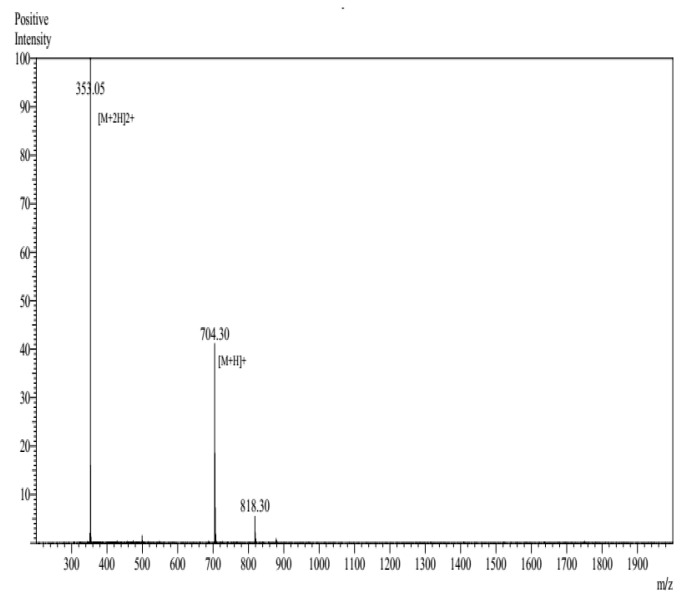
MS image of LA-RWR.

**Figure A2:**
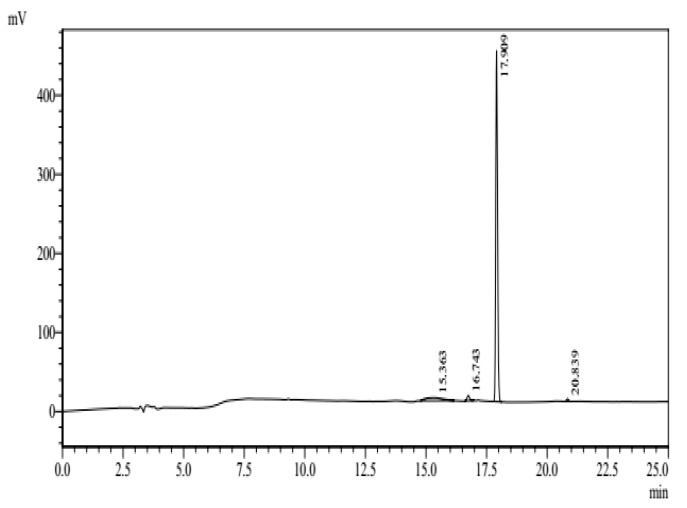
HPLC image of LA-RWR.

**Figure A3:**
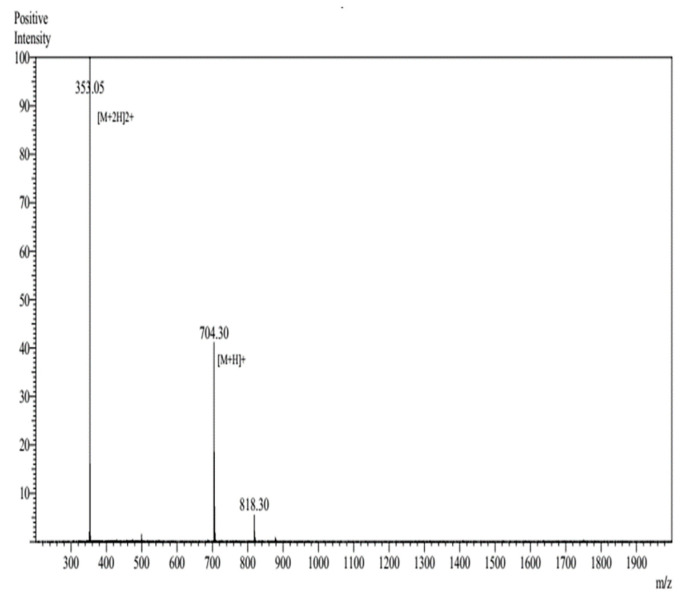
MS image of LA-RWR-PEA.

**Figure A4:**
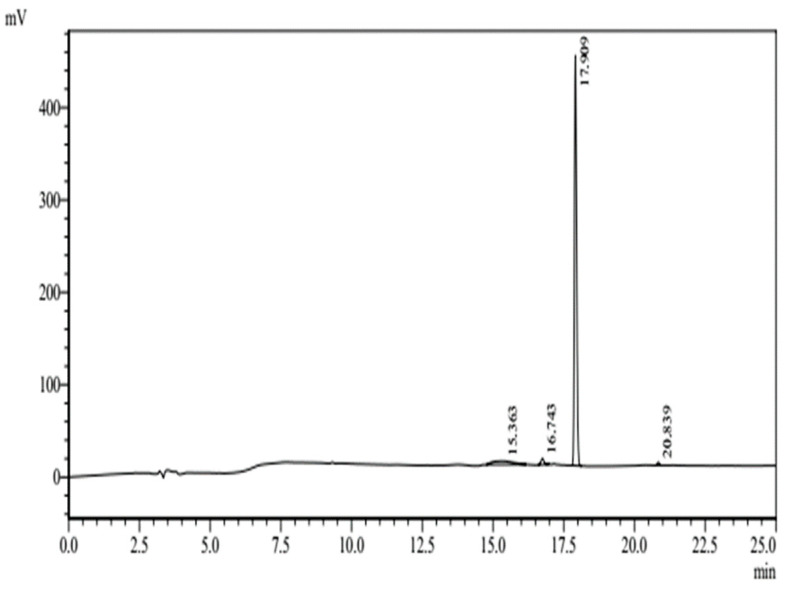
HPLC image of LA-RWR-PEA.

LA-RWR was synthesized using an Fmoc solid-phase synthesis method. The 2-chlorotrityl resin was subjected to a peptide synthesizer and extended using a carbodiimide-based activation following an initial Fmoc-deprotection. LA was treated as the fourth amino acid. After being cleaved from the resin, LA-RWR was modified by PEA on the C-terminus via DCC/HOBt coupling. The final products were purified using reverse-phase high-performance liquid chromatography (HPLC). All lipopeptides were analyzed and confirmed via HPLC and electrospray ionization mass spectrometry (ESI-MS) (see Figure A1, Figure A2, Figure A3 and Figure A4 in Appendix A for details). The molecular ion peaks of LA-RWR/LA-RWR-PEA were 704.81/808.4, which were consistent with the theoretical molecular weight, and the purities were 90.4%/90.7%.

### 2.3. Crosslinking of AMPs

**Figure 1:**
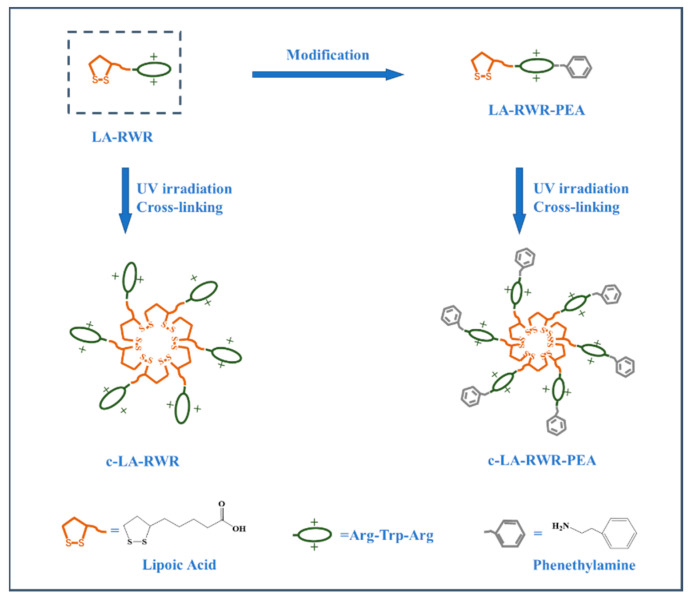
Schematic diagram of engineered ultra-short peptide nanoparticles with excellent antimicrobial activity.

LA-RWR and LA-RWR-PEA lipopeptides were prepared at 1 mg/mL in PBS buffer. The 1 mL LA-RWR solution was exposed to UV light with an intensity of 50 mW/cm^2^ for 40 min, while stirring with a magnetic stirrer (100 r/min). Crosslinked LA-RWR nanoparticles (c-LA-RWR) can be crosslinked via LA ring-opening under UV irradiation. A similar procedure was carried out for LA-RWR-PEA nanoparticles (c-LA-RWR-PEA) (Figure 1).

### 2.4. Characterization of AMP Nanoparticles

The morphological characteristics and sizes of crosslinked peptides nanoparticles were observed via transmission electron microscopy (TEM). A 2.0 μL volume sample was taken on the copper mesh carbon support film and observed via TEM (JEOLJEM2100). The average particle size and size distribution of c-LA-RWR and c-LA-RWR-PEA were determined using a ZS90 dynamic light scattering (DLS) device (Malvern, UK). Each sample was repeated at least 3 times, and all the experiments were performed at 25 °C.

### 2.5. Minimum Inhibitory Concentration (MIC) Assay

The antibacterial activities of LA-RWR, c-LA-RWR, LA-RWR-PEA, and c-LA-RWR-PEA were detected by measuring MIC using a broth microdilution assay. *E.* *coli* was proliferated overnight in LB culture medium and diluted to 10^6^ CFU/mL in PBS buffer. All peptide solutions were two-fold diluted with MHB medium from 512 to 0.5 μg/mL. A total of 125 μL of each dilution was dispensed into a 48-well microplate, followed by the addition of 125 μL of *E. coli* suspension. MHB served as a negative control and daptomycin was used as a control [26]. The MIC values were quantitated after incubation at 37 °C for 12 h by measuring the optical density at 540 nm. The method for determining the MIC values of peptides and nanoparticles against *S. aureus*, *C. albicans*, and MRSA was the same as mentioned above. The experiment was repeated at least 3 times.

### 2.6. Stability Evaluation

In order to evaluate the effects of LA-RWR, c-LA-RWR, LA-RWR-PEA, and c-LA-RWR-PEA on the bacteriostatic activity of *E. coli* at different values of pH (pH 6.8, pH 7.4, and pH 8.0), salt (100 mM NaCl, 1 mM CaCl_2_, and 1 mM MgCl_2_), and serum (5%), the MIC values under different conditions were determined [27]. Each experiment was repeated 3 times.

### 2.7. Time-Kill Kinetic Assay

The planktonic killing kinetics of LA-RWR, c-LA-RWR, LA-RWR-PEA, and c-LA-RWR-PEA were tested against *E.* *coli*. *E.* *coli* was grown in LB culture medium and diluted in PBS buffer to 10^7^ CFU/mL. Then, LA-RWR, c-LA-RWR, LA-RWR-PEA, and c-LA-RWR-PEA were each separately added to 10 mL of the bacterial suspension at a final concentration of 2MIC, and shaken at 200 rpm at 37 °C. The killing kinetics of planktonic bacteria was assayed after 6, 15, 30, 60, 120, and 240 min. At each predetermined time point, 100 μL of bacterial suspension was spread on the surface of agar plates, incubated overnight, and counted. The same method was used to test *S.* *aureus*, *C. albicans*, and MRSA. The experiment was repeated at least 3 times.

### 2.8. Anti-Biofilm Assay

*E.coli* was grown in LB medium to a concentration of 10^7^ CFU/mL, placed in 24-well plates, and incubated at 37 °C for 48 h to form biofilms. The plates were then washed three times with PBS. An added volume of 1 mL LB culture containing the peptide at a concentration of 2MIC and 1 mL LB was used as the control, and the incubation was continued for 5 h. Previous studies have indicated that biofilm biomass can be assessed using crystal violet (CV) staining [28,29,30]. The supernatant of the biofilm was discarded, washed, and then fixed in methanol for 20 min and stained with 1 mL of 0.1% CV solution for 5 min. Finally, 1 mL of 33% acetic acid solution was added to release the bound CV, and the absorbance was detected at 590 nm using a microplate reader.

The performance of the peptide to curb bacterial biofilm establishment was also investigated. Briefly, a mixture of *E. coli* (10^7^ CFU/mL) and peptides (2MIC) was added to a 48-well plate and incubated at 37 °C for 48 h. LB served as a control, and the biofilm density was calculated as follows: Biofilm density (%) = [(Abs_590nm_ in the peptides solution − Abs_590nm_ in LB)/(Abs_590nm_ in *E. coli* − Abs_590nm_ in LB)] × 100. The experiment was repeated at least 3 times for each well. The content determination method for the bacterial biofilm was the same as above. *S.* *aureus* and MRSA were tested using the same method.

In addition, bacterial biofilms were stained using the LIVE/DEAD™ BacLight™ Bacterial Viability Kit from KeyGEN Biotechnology, and the stained bacterial biofilms were subsequently observed via confocal laser scanning microscopy (CLSM) [31,32,33]. After 48 h, the formed biofilms were incubated with LA-RWR, c-LA-RWR, LA-RWR-PEA, and c-LA-RWR-PEA at MIC concentration for 5 h at 37 °C. Then, 1 μL of mixed fluorescent dyes was added and the biofilms were incubated at 37 °C for 15 min, placed on a slide, and observed via CLSM (Olympus, FV3000) at an excitation wavelength of 480 nm.

### 2.9. Membrane Depolarization Studies

The depolarization capabilities of peptides and nanoparticles on the cell membrane were investigated using the membrane potential-sensitive dye diSC_3_5 [34,35]. An overnight culture of *E. coli* DC2 was collected, washed with HEPES buffer, and diluted to 10^7^ CFU/mL. DiSC_3_5 was added to the bacterial solution to a final concentration of 0.4 μM, and incubated for 30 min. Then, 4 mM potassium chloride solution was added and incubated for another 10 min. LA-RWR, c-LA-RWR, LA-RWR-PEA, and c-LA-RWR-PEA were added to the mixture separately. Fluorescence changes were recorded within 10 min using a microplate reader (Spectra Max M5, Molecular Devices) with excitation and emission wavelengths of 622 and 670 nm. Bacteria incubated with diSC_3_5 served as the positive control, and each group of experiments was repeated at least 3 times.

### 2.10. Membrane Permeability Studies

The outer membrane permeabilities of peptides and nanoparticles were investigated using NPN [34,36]. *E. coli* was incubated overnight in LB medium and diluted to 10^7^ CFU/mL with PBS buffer. NPN solutions with bacterial suspensions were prepared at a concentration of 10 μM. LA-RWR, c-LA-RWR, LA-RWR-PEA, and c-LA-RWR-PEA were each separately added to the bacterial suspension at peptide concentrations of 2MIC. The fluorescence intensity was measured at excitation (350 nm) and emission (429 nm) wavelengths within 10 min on a fluorescence microplate reader (Spectra Max M5, Molecular Devices). Each group of experiments was repeated at least 3 times.

### 2.11. Visual Analysis

*E. coli* and *S. aureus* suspensions at a concentration of 10^8^ CFU/mL were each incubated with LA-RWR, c-LA-RWR, and LA-RWR-PEA for 30 min, then fixed for 12 h with 2.5% glutaraldehyde, and an ethanol gradient dehydration. Morphological changes before and after the peptides and nanoparticles treatment were observed using a scanning electron microscope (SEM, Fei Inspect F50) [37,38].

### 2.12. Hemolytic Activity Assay

To assess the biocompatibility of peptides and nanoparticles, we used rabbit erythrocytes as a cell model [39,40]. Here, 2.5 mL of rabbit blood was washed with normal saline at least three times and resuspended in 50 mL of normal saline to obtain 5% of blood cells. Different concentrations (from 512 to 2 μg/mL) of LA-RWR, c-LA-RWR, LA-RWR-PEA, and c-LA-RWR-PEA were incubated with the same volume of 5% blood cells at 37 °C for 3 h. Then, the mixture was collected via centrifugation (1000 rpm, 15 min). The UV-vis absorbance of the supernatant at 545 nm was recorded using a microplate reader (Model 680, Bio-Rad, CA, United States). The hemolysis rate was calculated as follows: Hemolysis (%) = [(Abs_545nm_ in the peptide solution − Abs_545nm_ in normal saline)/(Abs_545nm_ in distilled water − Abs_545nm_ in normal saline)] × 100. The experiment was repeated at least 3 times. 

### 2.13. Cytotoxicity Assay

The in vitro cytotoxicities of LA-RWR, c-LA-RWR, LA-RWR-PEA, and c-LA-RWR-PEA were investigated using the tetrazolium salt colorimetric assay [41]. Mouse fibroblasts (L929) and hamster kidney cells (BHK-21) were implanted separately into 48-well plates at 1 × 10^5^ cells per well for 24 h, and the cells were treated with peptides and nanoparticles for an additional 24 h. Subsequently, 32 μL of MTT solution was added to each well and incubated for another 4 h. After removing the supernatant, 100 μL of DMSO was added. Finally, the absorbance at 490 nm was measured with a microplate reader. The cell viability rate was calculated as follows: Cell viability (%) = [(Abs_490nm_ in the peptides solution − Abs_490nm_ in PBS)/ (Abs_490nm_ in culture medium − Abs_490nm_ in PBS)] × 100. Each experiment was repeated 3 times.

## 3. Results

### 3.1. Characterization of c-LA-RWR/c-LA-RWR-PEA Nanoparticles

**Figure 2:**
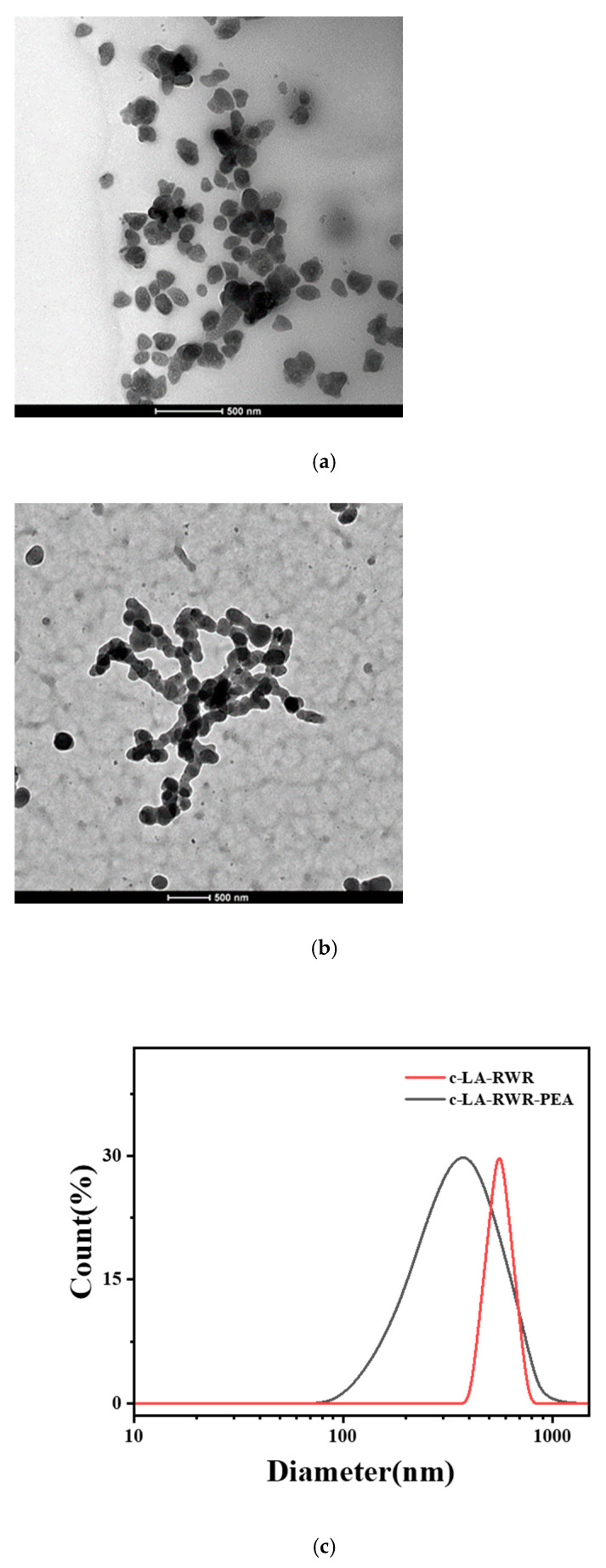
TEM images (**a**,**b**) and DLS (**c**) of c-LA-RWR and c-LA-RWR-PEA nanoparticles. The peptide concentration used for crosslinking was 1 mg/mL.

The morphology characteristics of c-LA-RWR/c-LA-RWR-PEA were confirmed via TEM, revealing the formation of nanoparticles with average sizes of 150/100 nm after crosslinking, as expected (see Figure 2a,b). Furthermore, DLS data confirmed that the hydrated particle sizes of c-LA-RWR/c-LA-RWR-PEA were approximately 390/550 nm (Figure 2c).

### 3.2. Antimicrobial Activities of Peptides in Vitro

The in vitro antimicrobial activities of peptides and nanoparticles were investigated by measuring the MIC values, involving *E.* *coli* (representative of Gram-negative bacteria), *S.* *aureus* (representative of Gram-positive bacteria), *C.* *albicans* (representative of fungi), and MRSA (representative of drug-resistant bacteria). Unexpectedly, the antibacterial activity of LA-RWR was not ideal, with MIC values ranging from 128 to 256 μg/mL, as shown in Table 1. Then, we detected the MIC values of c-LA-RWR, LA-RWR-PEA, and c-LA-RWR-PEA. Surprisingly, c-LA-RWR/LA-RWR-PEA/c-LA-RWR-PEA had excellent antibacterial activities compared to LA-RWR, with 64/128/256 times lower MIC values against *E.* *coli*. Likewise, the antibacterial effects of c-LA-RWR/LA-RWR-PEA/c-LA-RWR-PEA were 32/64/128, 16/16/32, and 16/32/64 times higher than those of LA-RWR for *S. aureus*, *C. albicans*, and MRSA, respectively.

The MIC values of LA-RWR, c-LA-RWR, LA-RWR-PEA, and c-LA-RWR-PEA for *E. coli* under different media conditions are listed in Table 2. In the presence of salt (100 mM NaCl, 1 mM CaCl_2_, and 1 mM MgCl_2_), the MIC values of lipopeptides and nanoparticles increased 2- or 4- fold compared with that of the control (pH 7.4, without salt). The serum stability was evaluated and the MICs were only increased by 2-fold in 5% serum. In addition, the MICs remained the same in pH 8.0 while they increased by 2-fold for *E. coli* in pH 6.8. The results indicate that the antibacterial activity of lipopeptides and nanoparticles was slightly reduced in lower pH and in the presence of serum. The effect of physiological salt ions on the stability of lipopeptides was similar to that of AMPs due to antagonistic effects [27].

**Figure 3:**
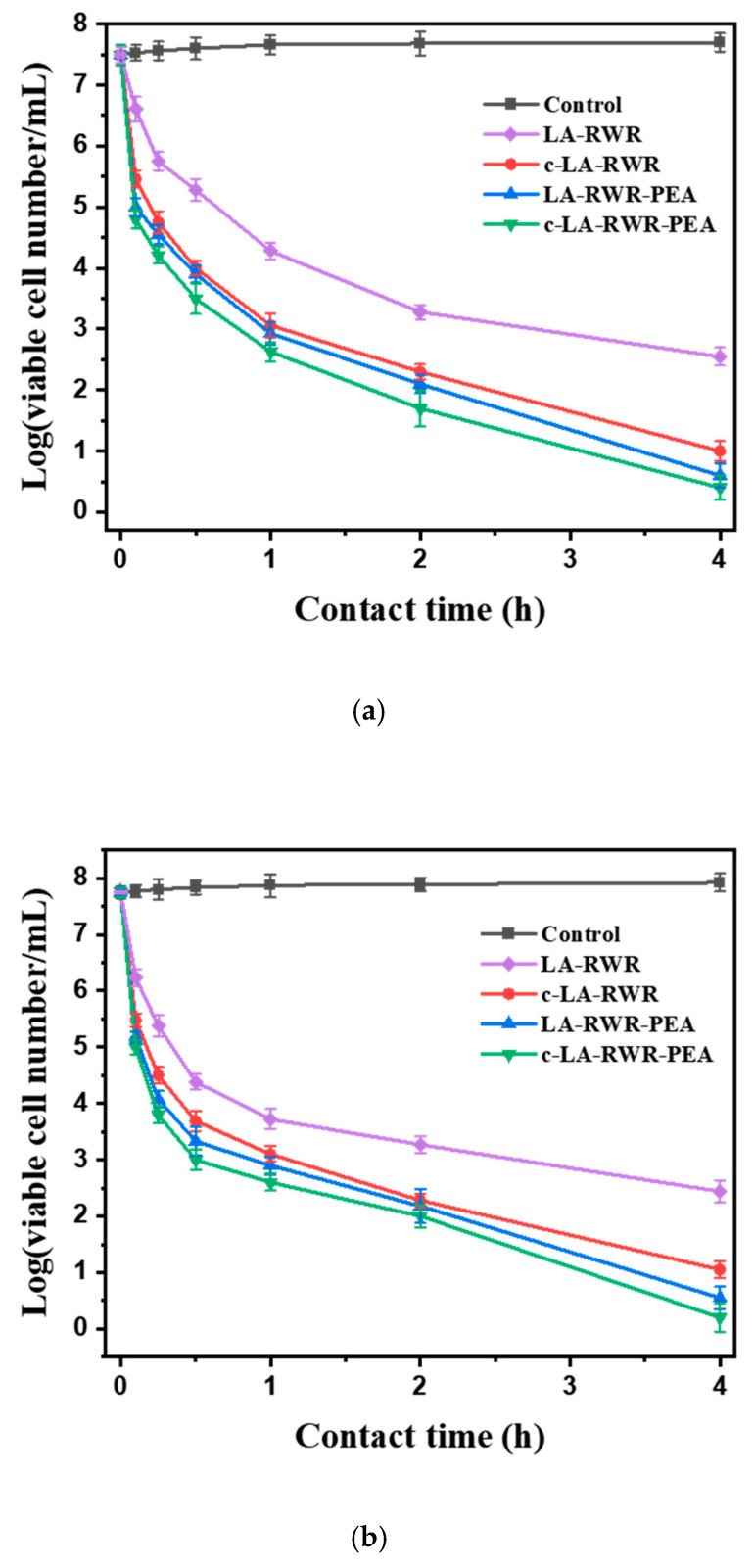

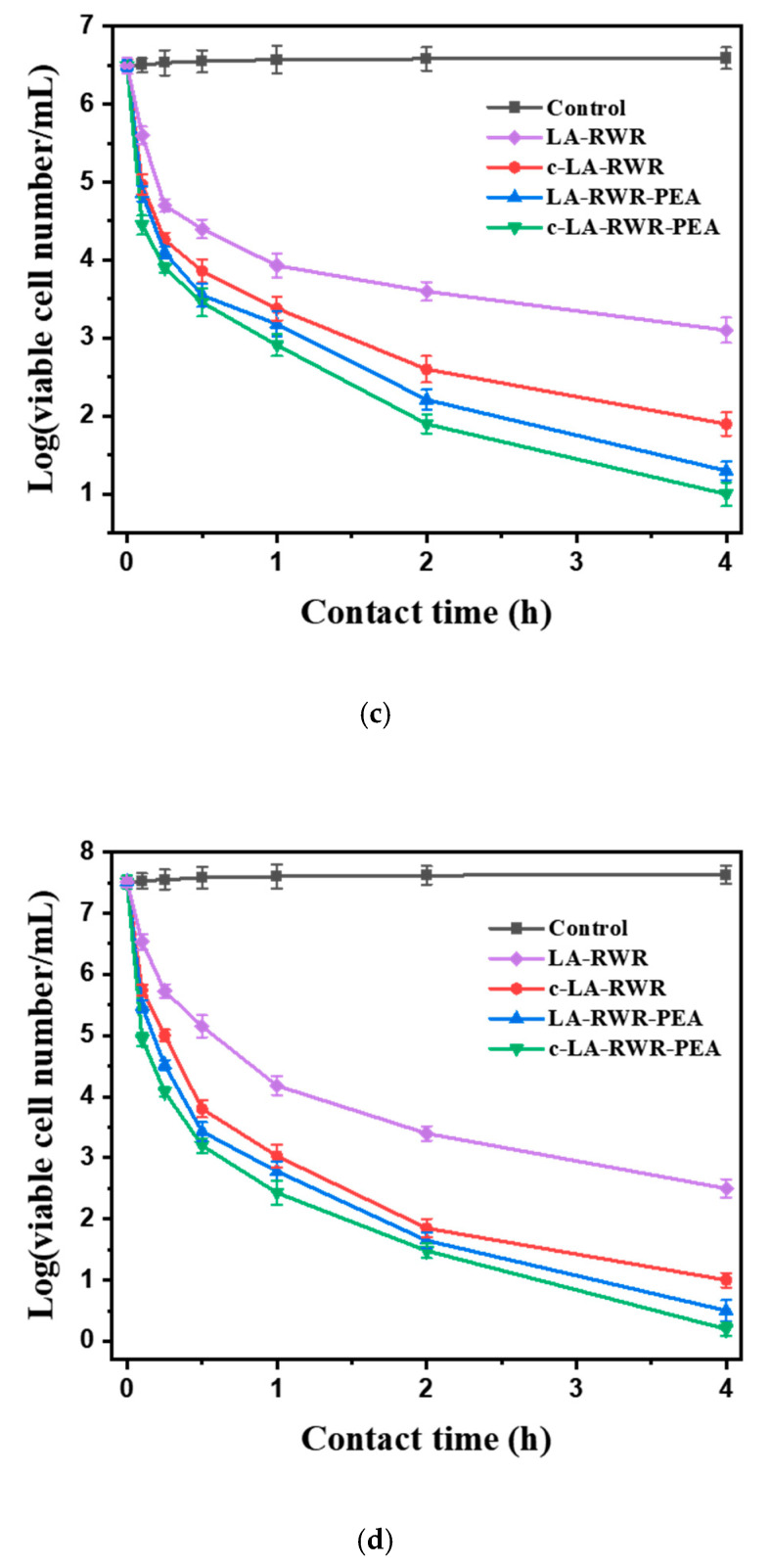
Time-kill curves of LA-RWR, c-LA-RWR nanoparticles, LA-RWR-PEA, and c-LA-RWR-PEA nanoparticles against (**a**) *E. coli*, (**b**) *S. aureus*, (**c**) *C. albicans*, and (**d**) MRSA.

The antibacterial kinetics of peptides and nanoparticles were evaluated using the plate coating method. The viabilities of bacteria (*E.* *coli*, *S.* *aureus*, MRSA, and *C.* *albicans*) co-cultured with LA-RWR, c-LA-RWR, LA-RWR-PEA, and c-LA-RWR-PEA were recorded over a series of time points. As is shown in Figure 3, the bactericidal effect of LA-RWR with a concentration of 2MIC (512 μg/mL) on *E.* *coli* was still poor, and the number of bacteria still exceeded 10^3^ CFU/mL after 4 h. However, when treated with c-LA-RWR/ LA-RWR-PEA at a concentration of 2MIC (8/4 μg/mL) for 4 h, the logarithm of the killing value decreased from 7.5 to 1.5/0.5; these results were significantly better than those for LA-RWR. Similar results were obtained for *S. aureus*, *C. albicans*, and MRSA.

### 3.3. Biofilm Eradication and Inhibition Activities of Peptides

The formation of bacterial biofilms does not involve a random accumulation of bacteria, but a highly differentiated group formed via density sensing, and the bacteria within the membrane can communicate through chemical signals. As a result, the susceptibility of bacteria in the biofilm to antibiotics is significantly lower than that in the free state, and the minimum susceptibility can be reduced by 1000-fold [42]. The antibacterial biofilm activities of the peptides and nanoparticles were explored, including the elimination of the formed biofilm and the inhibition of biofilm formation.

**Figure 4:**
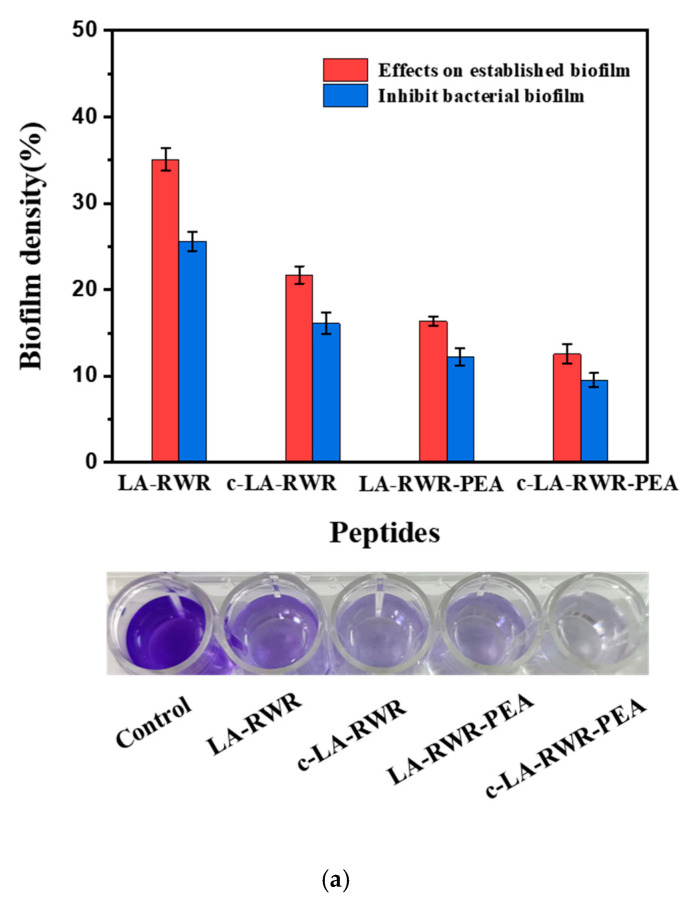

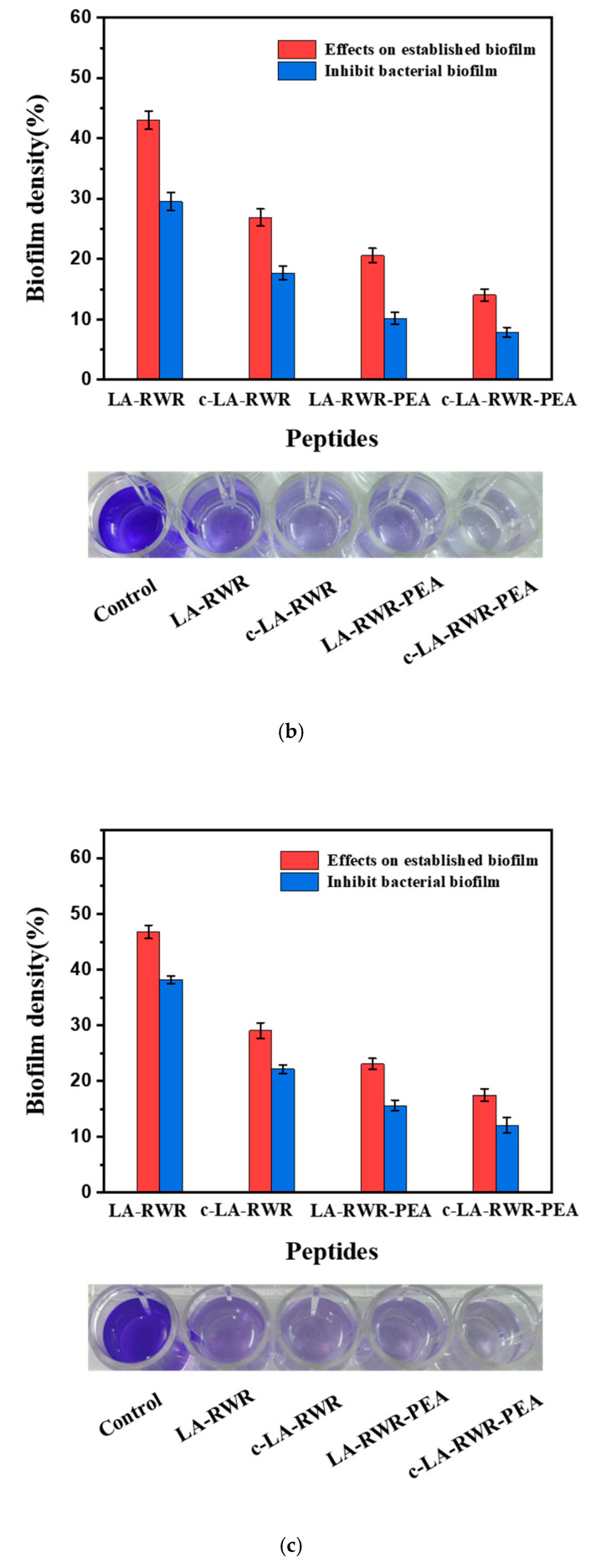
Effects of LA-RWR, c-LA-RWR nanoparticles, LA-RWR-PEA, and c-LA-RWR-PEA nanoparticles on the biofilm activities of (**a**) *E. coli*, (**b**) *S. aureus*, and (**c**) MRSA. Bacteria were grown in medium to a concentration of 10^7^ CFU/mL, placed in 24-well plates, and incubated at 37 °C for 48 h to form biofilms.

As is shown in Figure 4 (red column), when LA-RWR at a concentration of 2MIC (512 μg/mL) interacted with an *E. coli* biofilm for 5 h, the biofilm content decreased to 35%. However, the biofilm clearance rates of c-LA-RWR and LA-RWR-PEA at 2MIC (8/4 μg/mL) were 80% and 75% respectively, higher than those in the LA-RWR group. As is shown in Figure 4 (blue column), the bacterial biofilm contents were close to 15%/10% after c-LA-RWR/LA-RWR-PEA at 2MIC was co-incubated with *E. coli* for 48 h, whose values were higher than those of LA-RWR, with an inhibiting rate of more than 80%. Similarly, c-LA-RWR/LA-RWR-PEA showed a stronger clearance and inhibition rate than LA-RWR against *S. aureus* and MRSA.

**Figure 5:**
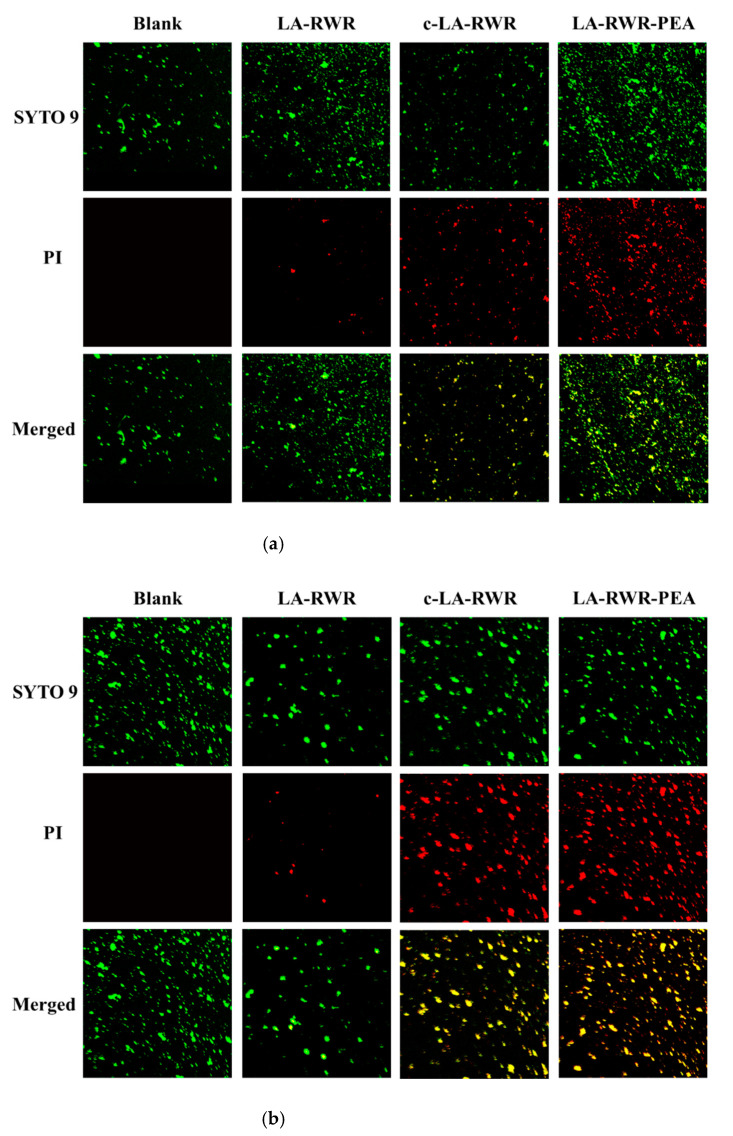
CLSM images of (**a**) *E. coli* and (**b**) *S. aureus* biofilms treated with LA-RWR, c-LA-RWR nanoparticles, and LA-RWR-PEA at 2MIC concentration for 5 h, respectively.

*E. coli* and MRSA biofilms were observed using CLSM (shown in Figure 5). SYTO 9 was able to stain live bacteria (green fluorescence) and can penetrate all cell membranes, while the other component, PI, was applied to stain dead bacteria (red fluorescence) and penetrate membrane permeability changes or dead bacteria. As is shown in Figure 5, LA-RWR at a concentration of 2MIC (512 μg/mL) exhibited little red fluorescence, indicating that the bacterial biofilm was still intact. However, c-LA-RWR and LA-RWR-PEA at 2MIC (8/4 μg/mL) showed more red fluorescence against *E. coli* and MRSA, revealing that a large number of biofilms were destroyed, and that the bacteria in the biofilm were damaged to varying degrees.

### 3.4. Antimicrobial Mechanisms of Peptides

#### 3.4.1. Mechanisms of Membrane Depolarization

To further understand the antibacterial mechanisms of LA-RWR, c-LA-RWR, and LA-RWR-PEA, we examined their effects on bacterial membrane potential. *E. coli* DC2 is an immunodeficient bacterium whose membrane potential can be maintained in a hyperpolarized state at high concentrations of K^+^. As a membrane-potential-sensitive probe, DiSC_3_5 accumulated in the hyperpolarized cell membrane and caused self-quenching. When peptides depolarized the membrane, the potential changed, and then the release of DiSC_3_5 into the solution caused fluorescence enhancement, which was proportional to the reduction in potential.

**Figure 6:**
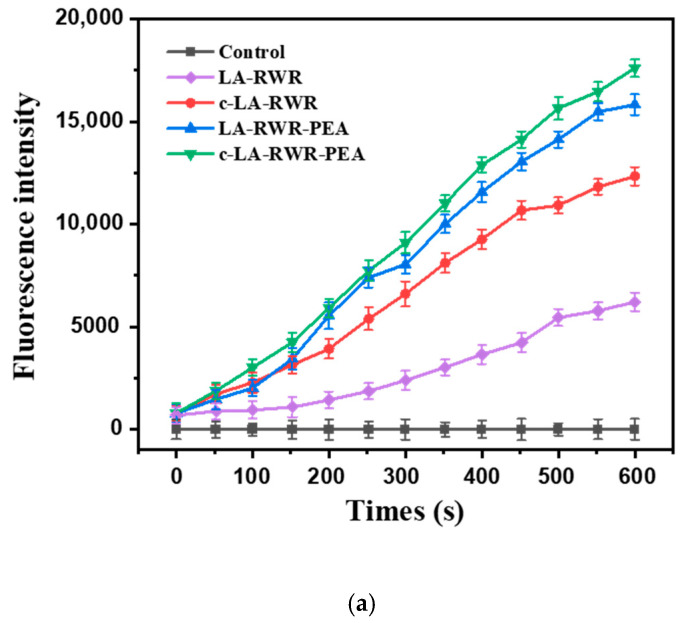

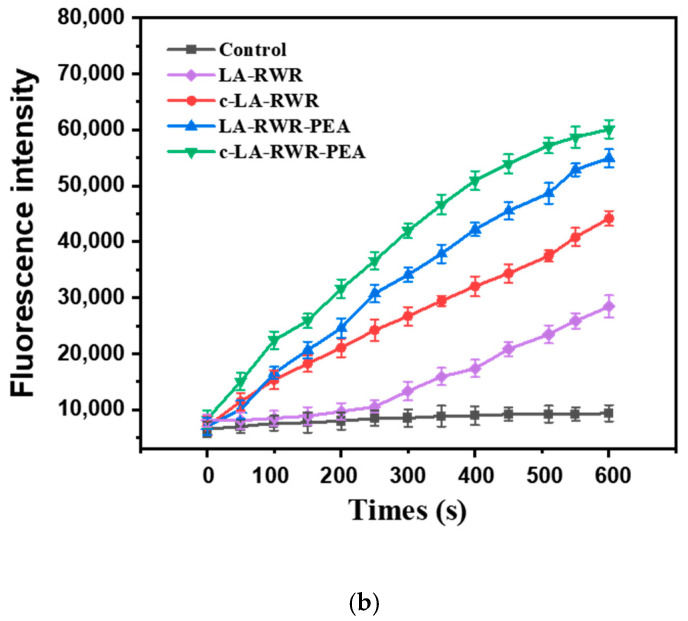
(**a**) Cytoplasmic membrane depolarization and (**b**) outer membrane permeability of *E. coli* treated with LA-RWR, c-LA-RWR nanoparticles, LA-RWR-PEA, and c-LA-RWR-PEA nanoparticles at 2MIC concentration for 10 min, respectively.

Figure 6a shows the change in fluorescence intensity within 10 min after adding LA-RWR, c-LA-RWR, LA-RWR-PEA, and c-LA-RWR-PEA. The fluorescence intensity of LA-RWR at 2MIC (512 μg/mL) increased slowly, with almost no fluorescence in the first 100 s, but the fluorescence levels of c-LA-RWR and LA-RWR-PEA at 2MIC (8/4 μg/mL) increased rapidly to much higher levels than the LA-RWR group; in addition, the highest fluorescence intensity of LA-RWR was less than half that of the c-LA-RWR group, and one-third that of the LA-RWR-PEA group.

#### 3.4.2. Mechanisms of Outer Membrane Permeability

The effect of peptides on bacterial outer membrane (OM) permeability were investigated using the fluorescent dye NPN. NPN fluoresces very weakly in aqueous solutions, but it strongly fluoresces in hydrophobic solutions such as *E. coli* lipid bilayers. When the cell membrane is disrupted by peptides, a channel is formed and NPN enters the hydrophobic environment to release strong fluorescence. Therefore, the change in the fluorescence intensity of the solution can reflect the permeability of the cellular OM (the strength of the destruction effect).

As is shown in Figure 6b, the fluorescence intensity of the peptides and nanoparticles increased with time, but the fluorescence intensity of LA-RWR at a concentration of 2MIC (512 μg/mL) was still weak. Surprisingly, c-LA-RWR/LA-RWR-PEA gradually reached stronger NPN fluorescence intensities with only 2MIC concentrations (8/4 μg/mL). Therefore, c-LA-RWR and LA-RWR-PEA disrupt the cytoplasmic membrane potential and also cause outer membrane rupture damage, both of which lead to the formation of channels that allow ions or larger molecules to pass through the membrane, resulting in cell death.

### 3.5. Visualization of Cell Damage

**Figure 7:**
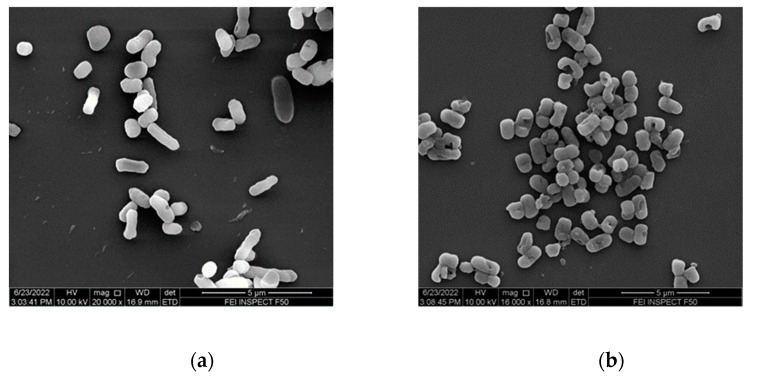

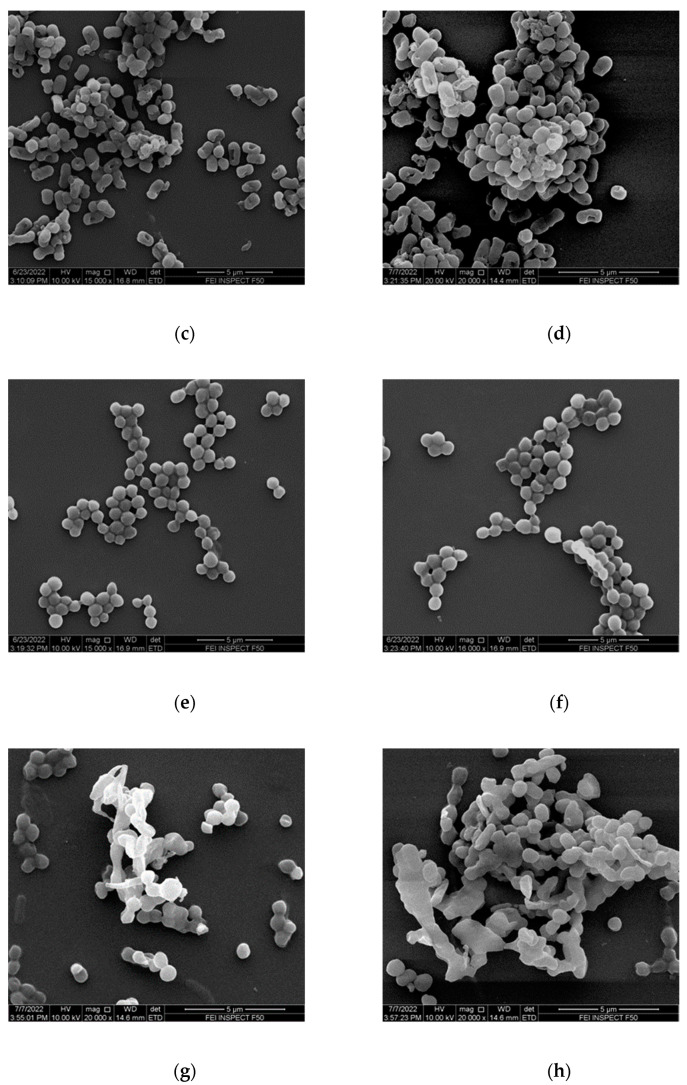
SEM images of *E. coli* (**a**–**d**) and *S. aureus* (**e**–**h**) that were untreated (**a**,**e**) and treated with LA-RWR (**b**,**f**), c-LA-RWR nanoparticles (**c**,**g**), and LA-RWR-PEA (**d**,**h**) at 2MIC concentration for 30 min, respectively.

The SEM images of *E. coli* and *S. aureus* morphology are shown in Figure 7. Visually, SEM images of untreated bacteria showed intact cells with typical morphologies and well-defined cell membrane boundaries (Figure 7a,e). Bacteria were co-cultured with LA-RWR for 30 min, and a slight shrinkage of the cell membrane was observed (Figure 7b,f). However, when *E. coli* and *S. aureus* were incubated with c-LA-RWR (Figure 7c,g)/LA-RWR-PEA (Figure 7d,h) for 30 min, the morphologies of almost all the bacteria were changed, including the appearance of numerous pores in the membrane and the outflow of cytoplasm.

### 3.6. In Vivo Safety Evaluation of Peptides

**Figure 8:**
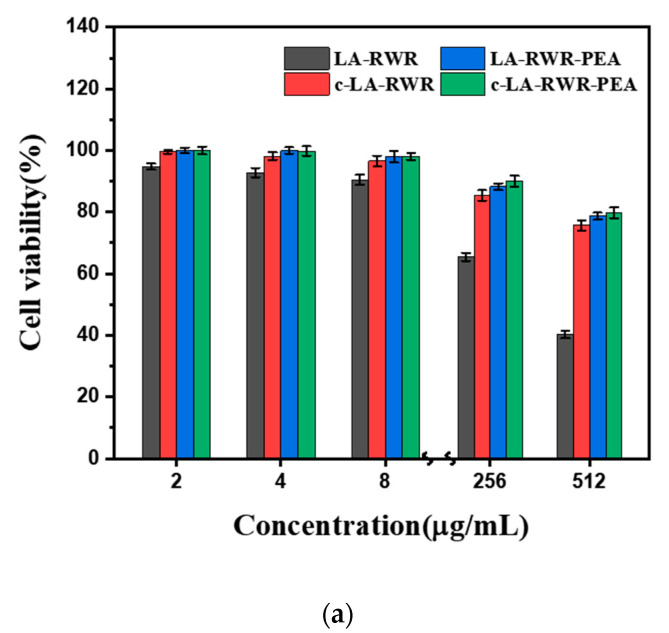

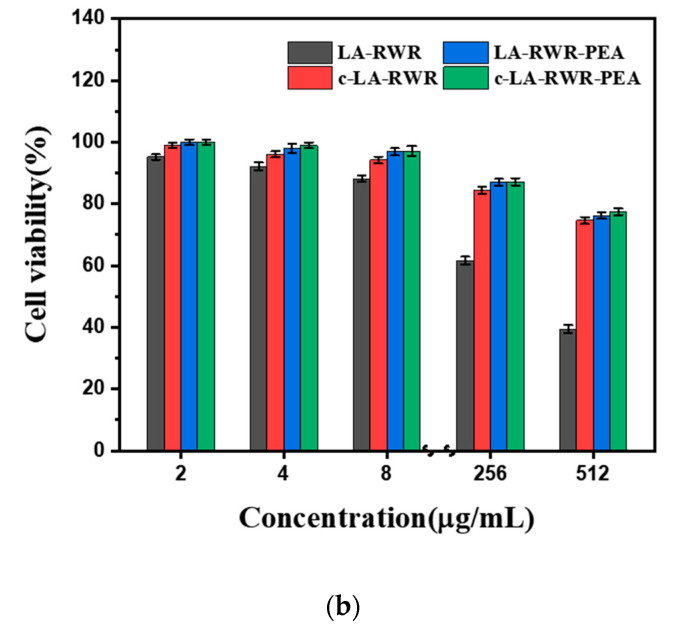
Cell viability of (**a**) L929 cells and (**b**) BHK-21 cells treated with LA-RWR, c-LA-RWR nanoparticles, LA-RWR-PEA, and c-LA-RWR-PEA nanoparticles.

The biosafety of LA-RWR, c-LA-RWR, LA-RWR-PEA, and c-LA-RWR-PEA was investigated, including hemolysis and cytotoxicity [43]. As is shown in Table 3, the hemolysis rate of LA-RWR at 2MIC (512 μg/mL) exceeded 5%, indicating that hemolysis occurred and that the toxicity was high. However, c-LA-RWR/LA-RWR-PEA did not display hemolysis at 2MIC (8/4 μg/mL). Figure 8 shows that LA-RWR had certain cytotoxicities against L929 and BHK-21 cells at the concentration of 2MIC (512 μg/mL) and the survival rate of the two kinds of cells was only 40%, indicating that LA-RWR has high cytotoxicity. However, when treated with c-LA-RWR/LA-RWR-PEA at a concentration of 2MIC (8/4 μg/mL), the cell viability was approximately 100% and the safety effect was better than that of LA-RWR.

## 4. Discussion

The MIC results indicate that the antibacterial activity of LA-RWR was greatly enhanced after crosslinking and C-modification with PEA. According to the literature reports, we speculated that the nanoparticles studied in this paper were very likely to use their unique particle sizes to enter the phospholipid bilayer [44], thereby causing damage to the cell membrane and resulting in the contents flowing out of the channel, while the modification of PEA improved the hydrophobicity of LA-RWR, which was more conducive toward interaction with the cell membrane [45]. The plankton-killing kinetics proved that the in vitro antibacterial effect of c-LA-RWR/LA-RWR-PEA formed via crosslinking/C-modification was greatly enhanced, exhibiting antibacterial activities at extremely low concentrations. The anti-biofilm results show that c-LA-RWR and LA-RWR-PEA not only had a good killing effect on free bacteria, but also had a strong killing effect on highly differentiated biofilms. We speculate that the modified peptides can better disintegrate or inhibit the adhesion between bacteria, which is not conducive to the formation of biofilms [46]. The CLSM results reconfirm that both c-LA-RWR/LA-RWR-PEA can cause substantial damage to bacterial biofilms at low concentrations. The results of membrane depolarization show that ultra-low concentrations of c-LA-RWR/LA-RWR-PEA have significant depolarization effects on membrane depolarization. Similarly, the antibacterial mechanism study of c-LA-RWR/LA-RWR-PEA also confirmed that crosslinking/C-modification can accelerate bacterial membrane changes, which is consistent with the membrane destruction mechanism. SEM images of cell damage suggest that c-LA-RWR/LA-RWR-PEA can exert its bactericidal activity by physically disrupting the cellular structure to eventually lyse the entire cell, which is consistent with the membrane disruption mechanism reported in the literature [47]. Furthermore, it can be seen that c-LA-RWR/LA-RWR-PEA were obviously more selective in distinguishing between bacteria and human erythrocytes. Therefore, the biocompatibilities of c-LA-RWR, LA-RWR-PEA, and c-LA-RWR-PEA were qualified.

## 5. Conclusions

In summary, lipopeptides LA-RWR and LA-RWR-PEA were synthesized and crosslinked by ring opening of LA to form nanoparticles. LA-RWR-PEA, c-LA-RWR, and c-LA-RWR-PEA nanoparticles exhibited excellent antibacterial activities against *E. coli*, *S. aureus*, *C. albicans*, and MRSA, indicating at least a 16-fold reduction in MIC values compared with those of LA-RWR. The results of evaluation using planktonic and biofilm bacteria revealed that lipopeptide nanoparticles had faster bactericidal rates and higher clearance/inhibition effects on biofilms. In addition, the antibacterial mechanism of c-LA-RWR/LA-RWR-PEA was explored, which exhibited excellent membrane depolarization and outer-membrane permeabilization ability, disrupting the integrity of the cell membrane and eventually leading to the death of bacteria. A superior antimicrobial performance with good biosafety would make c-LA-RWR/LA-RWR-PEA good candidates for various antimicrobial applications.

As LA-RWR-PEA alone showed improved antibacterial performance, the MIC values were only decreased by 2-fold after crosslinking. No significant difference was revealed between LA-RWR-PEA and c-LA-RWR-PEA nanoparticles. Biosafety of the modified lipopeptides and crosslinked nanoparticles evaluated via in vivo assays would be considered in further study.

## Figures and Tables

**Table 1:** The MICs of LA-RWR, c-LA-RWR nanoparticles, LA-RWR-PEA, c-LA-RWR-PEA nanoparticles, and daptomycin as control.

Peptides	MIC (µg/mL)			
	*E. coli*	*S. aureus*	*C. albicans*	MRSA
LA-RWR	256	256	128	256
c-LA-RWR	4	8	8	16
LA-RWR-PEA	2	4	8	8
c-LA-RWR-PEA	1	2	4	4
Daptomycin	1	1	2	2

**Table 2:** Effects of pH, salt, and serum on antibacterial activities (in µg/mL) of LA-RWR, c-LA-RWR nanoparticles, LA-RWR-PEA, and c-LA-RWR-PEA nanoparticles against *E. coli*.

Peptides	pH 6.8	pH 7.4 (Normal Medium)	pH 8.0	100 mM NaCl	1 mM CaCl_2_	1 mM MgCl_2_	5% Serum
LA-RWR	512	256	256	512	512	512	256
c-LA-RWR	4	4	4	16	8	16	8
LA-RWR-PEA	2	2	2	8	4	8	4
c-LA-RWR-PEA	2	1	1	4	4	8	2

**Table 3:** Hemolysis rates of LA-RWR, c-LA-RWR nanoparticles, LA-RWR-PEA, and c-LA-RWR-PEA nanoparticles.

Peptides	Hemolysis Rate (%) Concentration (µg/mL)								
	512	256	128	64	32	16	8	4	2
LA-RWR	9.11	7.42	6.25	5.68	4.11	3.98	3.41	2.27	1.14
c-LA-RWR	5.45	5.03	4.68	4.27	3.11	2.7	1.68	1.14	0.57
LA-RWR-PEA	5.27	4.85	4.41	3.98	3.17	2.84	2.27	1.25	0.57
c-LA-RWR-PEA	4.63	3.7	3.11	2.84	2.27	1.7	1	0	0

## Data Availability

Not applicable.
